# Plasma renin activity profile of patients with congenital adrenal hyperplasia in Semarang, Indonesia: a preliminary study

**DOI:** 10.1186/1687-9856-2013-S1-P137

**Published:** 2013-10-03

**Authors:** Mahayu Dewi Ariani, Nani Maharani, Udin Bahrudin, Agustini Utari, Sultana MH Faradz

**Affiliations:** 1 Center for Biomedical Research (CEBIOR), Faculty of Medicine, Diponegoro University, Semarang, Indonesia; 2 Division of Endocrinology, Pediatric Department, Faculty of Medicine, Diponegoro University/ dr. Kariadi Hospital, Semarang, Indonesia

## 

Congenital Adrenal Hyperplasia (CAH) is an adrenal disorders due to impaired activity of one of the enzymes required for cortisol and aldosterone biosynthesis. One of the subtypes of CAH is the salt-wasting (SW) form which there is a renal salt loss due to aldosterone deficiency. Plasma Renin Activity (PRA) is the main index used to evaluate the mineralocorticoid control in CAH. PRA testing is almost very rare done for the CAH patients due to high cost, sophisticated laboratory is not available in all region and no compulsary health insurance in Indonesia. The objective of this research was to describe PRA level in patient with Congenital Adrenal Hyperplasia.

This study is a part of CAH cohort study in Center for Biomedical Research (CEBIOR), Semarang, Indonesia. Eighteen patients diagnosed as CAH were drawned blood samples for hormonal test, including 17 OHP, Plasma Renin Activity, Cortisol, and Electrolytes. Clinical history and physical examination was performed to each patients.

All 18 patients (17 female and 1 male) have high 17-OHP level and normal electrolites levels, but only 14 patients have PRA data. Four patients did not show their PRA level due to fail in too much blood collection. Out of 14 patient, four patients which had history of SW have very high PRA level, while six patients have PRA level more than normal without history of salt wasting. The mean of PRA level in patients with history of SW (47.72 (SD 18.63)) are higher than the patient without history of SW (13.97 (SD 13.3)).

This study suggest that PRA level might be useful for evaluating mineralocorticoid level in CAH. It is proposed to the government to subsidize and provide PRA and other hormonal testing for CAH in many regions with affordable cost.

